# Hereditary Tyrosinemia Type-1 With Late Presentation: A Case Report

**DOI:** 10.7759/cureus.62990

**Published:** 2024-06-23

**Authors:** Md Ilyaz, Renuka S Jadhav, Vineeta Pande, Shailaja Mane, Pranavi Mokkarala

**Affiliations:** 1 Pediatrics, Dr. D. Y. Patil Medical College, Hospital & Research Centre, Dr. D. Y. Patil Vidyapeeth (Deemed to be University), Pune, IND

**Keywords:** nodular cirrhosis, parenchymal disease, limb edema, autosomal recessive disorders, tyrosinemia type 1

## Abstract

The etiology of hereditary tyrosinemia type 1 (HT-1) is the absence of fumarylacetoacetate hydrolase (FAH), an enzyme that catalyzes the last stage of the tyrosine breakdown process. With an autosomal recessive inheritance pattern, it is an uncommon genetic condition. We present a case of HT-1 in a three-year-old female child characterized by abdominal distension, facial edema, lower limb edema, and an enlarged liver with parenchymal disease. A liver biopsy confirmed mixed nodular cirrhosis, and a subsequent whole exome sequencing revealed autosomal recessive inheritance of tyrosinemia type 1. The patient is currently undergoing treatment with capsule nitisinone 5 mg, which inhibits the second step of tyrosine degradation to prevent tyrosinemia, along with a restricted protein diet, while awaiting liver transplantation.

## Introduction

Hereditary tyrosinemia type 1 (HT-1) is a rare genetic disorder with autosomal recessive inheritance resulting from the lack of the enzyme fumarylacetoacetate hydrolase (FAH). FAH is indispensable for tyrosine degradation and is required to catalyze the final step of the degradation pathway [1,2]. Its prevalence is estimated at 1:100,000 [1,3]. HT-1 exhibits a heterogeneous clinical spectrum involving the renal, hepatic, or central nervous systems, with life-threatening consequences if left untreated [4]. Early mortality is common, with a heightened risk of hepatocellular carcinoma among survivors. Long-term complications of HT-1 encompass cirrhosis, characterized by scarring and poor liver function, as well as the development of liver nodules, which may progress to hepatocellular carcinoma. Additionally, individuals with HT-1 are prone to hypophosphatemic rickets, a condition marked by soft or weakened bones due to impaired phosphate regulation. Glomerulosclerosis, involving scarring of the kidney's filtering units, and chronic renal failure, leading to progressive loss of kidney function, are also observed as significant complications of HT-1 [5,6]. Early detection and management are essential to mitigate the impact of these long-term complications and improve patient outcomes. Despite advancements, unresolved issues persist regarding appropriate follow-up of cirrhotic patients, optimal timing of liver transplantation to prevent carcinoma, and the chronic renal disease's long-term evolution. Herein, we present a case of late-presenting HT-1.

## Case presentation

A three-year-old female child presented to our tertiary specialist unit with complaints of facial puffiness and lower limb edema for the past six months and abdominal distension, which was progressive in nature. She exhibited normal anthropometry and no history of fever, jaundice, or melena. There were no prior hospitalizations, and she had completed immunizations up to her age, achieving all developmental milestones up to the age of three. She is the second child born to third-degree consanguineous parents, with her other two siblings having no significant medical history. On physical examination, the child appeared active and revealed firm hepatomegaly and mild splenomegaly.

**Figure 1:**
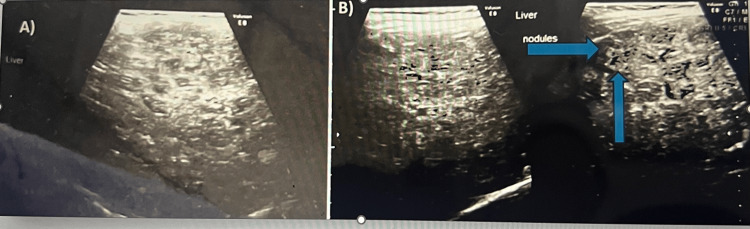
USG images showing (A) a heterogeneous echotexture and (B) multiple ill-defined nodules, with the largest measuring 1.1 × 1.2 cm (arrow marked) USG: ultrasound sonography

Abdominal ultrasonography revealed an enlarged liver with a nodular surface and heterogeneous echotexture, along with multiple ill-defined nodules, the largest measuring 1.1 × 1.2 cm (Figure 1). No focal mass, abscess, or intrahepatic biliary dilatation was observed, and there was no evidence of calcification. Mild splenomegaly was also noted. The kidneys appeared bulky and enlarged, with raised parenchyma suggesting a storage disorder. Routine blood investigations showed normal hemoglobin, leukocyte, and platelet counts, with a slightly elevated eosinophil count (Table 1). Serum tyrosine, methionine, and urine succinyl acetone levels were increased. The liver biopsy indicated mixed nodular cirrhosis with activity. Although progressive familial intrahepatic cholestasis (PFIC) type 3 could not be ruled out, the tandem mass spectrometry screening results were negative. Gas chromatography-mass spectrometry (GC-MS) screening was inconclusive, but increased excretion of tyrosine metabolites, hypoxanthine, and xanthine was noted. Whole exome sequencing identified *FAH* gene mutation consistent with tyrosinemia type 1.

**Table 1:** Blood examination VBG: venous blood gas; pCO_2_: partial pressure of carbon dioxide; pO_2_: partial pressure of oxygen; HCO_3_: bicarbonate; Hb: hemoglobin; WBC: white blood cells; RBC: red blood cells; PCV: packed cell volume; MCV: mean corpuscular volume; MCH: mean corpuscular hemoglobin; MCHC: mean corpuscular hemoglobin concentration; RDW: red cell distribution width

Parameter	Observed Value	Reference Value
VBG-pH	7.396	7.35-7.45
pCO_2_	35.5	35-45
pO_2_	46.2	83-108
HCO_3_	21.3	21-28
Lactic acid	1.7	<2
Hb	12.2 g/dL	13.5-22 g/dL
WBC	9700	4000-11000
Neutrophils	38.1%	40-80%
Platelets	136000/uL	150000-415000/uL
Lymphocyte	41%	20-40%
Eosinophils	5.3%	1-6%
Basophils	0.7%	0.2%
Monocytes	6.7%	-
RBC count	4.97 x 10^6^/μL	4.10-5.30 x 10^6^/μL
PCV	38.20%	40-50%
MCV	76.9	80-100
MCH	24.50	27-31
MCHC	31.90	32-26
RDW	30.30%	12-14.5%
Sodium	136 mEq/L	135-145 mEq/L
Potassium	3.9 mmol/L	3.5-4.5 mmol/L
Chloride	95 mEq/L	98-107 mEq/L
Phosphorus	3.60 mg/dL	2.6-4.7 mg/dL
Ionic calcium	5.281 mg/dL	4.8-5.5 mg/dL

The child was initiated on capsule nitisinone 5 mg, which inhibits the second step in the tyrosine degradation pathway, thus preventing tyrosine accumulation in the bloodstream due to the deficiency of the FAH enzyme. Additionally, the child was administered the Tyros-1 formula. Liver transplantation remains the cornerstone of treatment for this disease. Presently, the child is being managed medically.

## Discussion

There are three distinct forms of tyrosinemia. Type 1 is characterized by neurological crises, progressive liver disease, an increased risk of hepatocellular carcinoma, and renal tubular dysfunction. Additionally, hypophosphatemic rickets is a common feature. Acute hepatic failure due to micro- and macronodular cirrhosis manifests before six months of age, while subacute cases presenting after six months are marked by rickets, hepatomegaly, and bleeding abnormalities. A US and Canadian consensus group review and recommendations on the diagnosis and treatment of HT-1 have attributed this disorder to hepatic failure and comorbidities affecting the renal and neurological systems. The study further asserts that patients with HT-1 also face long-term risks for developing hepatocellular carcinoma due to a deficiency in FAH and, thereby, the accumulation of toxic metabolites, causing progressive liver and kidney damage and neurological crises [7]. Another study by Maria et al. (2023) reports that the manifestations of tyrosinemia type I begin in the first month of life, with the earliest and most significant impact on the liver. Infants typically present with symptoms such as jaundice, hepatomegaly, and elevated liver enzymes, reflecting the profound hepatic dysfunction that characterizes the early stages of the disease [8].

Chronic cases, appearing after one year, involve growth retardation, rickets, and hepatomegaly. Tyrosinemia type II, or oculocutaneous tyrosinemia, results from a deficiency of hepatic tyrosine aminotransferase [9]. Clinical manifestations include hyperkeratotic lesions on the digits, palms, and soles, ocular ulcerations, and cognitive and motor impairment [8]. Type III tyrosinemia is caused by a deficiency of the enzyme 4-hydroxyphenylpyruvate dioxygenase, with patients presenting with growth retardation, seizures, and ataxia. The most distinguishing feature of type I tyrosinemia is liver and kidney involvement, with elevated levels of urine succinylacetone and serum tyrosine and methionine being key diagnostic markers [5].

Standard treatment includes tyrosine- and phenylalanine-restricted diets, with liver transplantation proving successful for many patients. Another therapeutic approach involves the use of NTBC (nitisinone), which inhibits 4-hydroxyphenylpyruvate dehydrogenase, an enzyme in the tyrosine metabolism pathway. A study involving 32 patients with type I tyrosinemia reported findings such as aminoaciduria (82%), hypercalciuria (67%), tubular acidosis (59%), nephromegaly (47%), renal hyperechogenicity (47%), nephrocalcinosis (16%), and reduced glomerular filtration rate (48%) [10]. In another study of eight patients, nephromegaly, tubulopathy, and vitamin D-resistant rickets were observed in 50%, 80%, and 50% of cases, respectively [11]. The toxic agent in type I tyrosinemia is succinylacetone, and NTBC treatment prevents its accumulation, significantly reducing the risk of hepatocarcinoma [12]. Diagnosis is confirmed through DNA mutation analysis, with mutations in the *FAH* gene on exon 12 (OMIM#276700) indicating autosomal recessive inheritance.

## Conclusions

To reduce or completely prevent the severe consequences of HT-1, such as progressive liver failure, renal dysfunction, hypophosphatemic rickets, severe neurological crises, and the early onset of hepatocellular carcinoma (HCC), early diagnosis is crucial. This can be achieved by initiating nitisinone (NTBC) treatment before one month of age and implementing universal newborn screening for tyrosinemia.

Early diagnosis and prompt initiation of nitisinone therapy, combined with dietary management, typically lead to better patient outcomes. Prompt administration of nitisinone, widespread neonatal screening, and appropriate, regular monitoring can halt disease progression and significantly improve the patient’s quality of life.
